# CAR T Cell Therapy for Pediatric Brain Tumors

**DOI:** 10.3389/fonc.2020.01582

**Published:** 2020-08-12

**Authors:** John D. Patterson, Jeffrey C. Henson, Rebecca O. Breese, Kevin J. Bielamowicz, Analiz Rodriguez

**Affiliations:** ^1^Department of Neurosurgery, University of Arkansas for Medical Sciences, Little Rock, AR, United States; ^2^Department of General Surgery, Wake Forest Baptist Medical Center, Winston-Salem, NC, United States; ^3^Division of Hematology/Oncology, Department of Pediatrics, Arkansas Children’s Research Institute, Little Rock, AR, United States

**Keywords:** chimeric antigen receptor T cell, pediatric brain tumor, medulloblastoma, ependymoma, ATRT, pediatric glioma, immune therapy, adoptive cell therapy

## Abstract

Chimeric Antigen Receptor (CAR) T cell therapy has recently begun to be used for solid tumors such as glioblastoma multiforme. Many children with pediatric malignant brain tumors develop extensive long-term morbidity of intensive multimodal curative treatment. Others with certain diagnoses and relapsed disease continue to have limited therapies and a dismal prognosis. Novel treatments such as CAR T cells could potentially improve outcomes and ameliorate the toxicity of current treatment. In this review, we discuss the potential of using CAR therapy for pediatric brain tumors. The emerging insights on the molecular subtypes and tumor microenvironment of these tumors provide avenues to devise strategies for CAR T cell therapy. Unique characteristics of these brain tumors, such as location and associated morbid treatment induced neuro-inflammation, are novel challenges not commonly encountered in adult brain tumors. Despite these considerations, CAR T cell therapy has the potential to be integrated into treatment schema for aggressive pediatric malignant brain tumors in the future.

## Introduction

Chimeric antigen receptor (CAR) T cells are a form of adoptive cell therapy used for immunotherapy. CAR T cells were initially FDA approved in 2017 for hematological malignancies, however, many preclinical and clinical studies have shown efficacy of CAR T cells for solid tumors including glioblastoma, medulloblastoma, and ependymoma (1–3). Pediatric central nervous system (CNS) cancers remain the leading cause of pediatric cancer related death, thus there is an urgent need to develop new therapies (4). These new therapies need to be specifically directed to malignant cells and limit off target cytotoxicity inherent in chemotherapeutics while having a strong, sustained cytotoxic effect on cancer cells to minimize recurrence. CAR-T cells have the potential to accomplish these goals. In this review, we discuss the current progress in CAR T cell development for specific pediatric brain tumors as well as future implementation strategies.

### Overview of CAR-T Cell Targeting

The concept of engineering chimeric antigen receptors (CAR) has been around for over 25 years, and entails combining a single-chain variable fragment (scFv) of an antibody with the T cell receptor (TCR) signaling domain CD3, thus conferring antibody like antigen recognition to T cell cytolytic activity (5, 6). This ingenuity allows for the recognition of a target antigen without presentation by major histocompatibility complex (MHC) (5). However, in order for the T cell to carry out its cytolytic activity, proliferate and maintain persistence in the local microenvironment, co-stimulation is still necessary (7). In nature these co-stimulatory signals are provided by the antigen presenting cell, but in engineered CAR T cell constructs multiple co-stimulatory domains can be included to promote T cell functionality (8–10).

For applications of CAR T cells in cancer treatment, the engineered target is ideally only present on the tumor and not on normal cells thus limiting off target therapeutic effects (1). CAR T cell therapy became FDA approved in 2017 for B cell malignancies by targeting CD19 (11). The applications for CAR T cells continues to expand in the clinical setting especially in the treatment of hematological malignancies (12). Multiple clinical trials are in place for evaluating the efficacy of CAR T cell therapy in solid tumors but to date CAR therapy for this indication is not FDA approved. Many obstacles are present that hinder the efficacy of CAR T therapy in solid tumors which includes but are not limited to difficulty in trafficking to the tumor site, presence of an immunosuppressive environment, toxicity, and tumor antigen heterogeneity (13).

**FIGURE 1:**
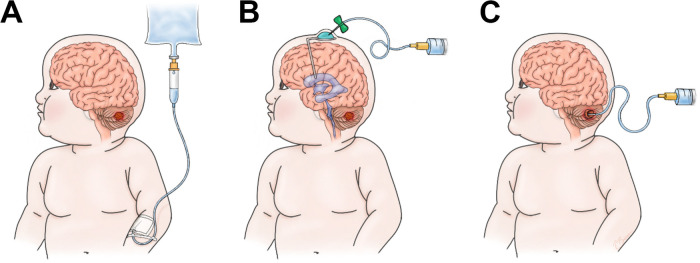
Options for infusion of CAR-T cells. **(A)** Intravenous (IV), **(B)** Intrathecal/Ventricular (IT), **(C)** Intratumor/cavity (IC).

Solid tumors in the brain present further difficulties due to the semipermeable properties of the blood brain barrier (BBB) which limits the delivery of many therapeutics (14). The BBB is comprised of specialized endothelial cells that prevent entry of large hydrophophilic molecules and unwanted cells from entering the brain. Brain tumors disrupt the BBB to form the blood- tumor barrier (BTB) which has heterogenous perfusion and permeability throughout the tumor and also hinders the delivery of therapeutics (15). CAR T cell infusion to the brain can include delivery via the blood, cerebral spinal fluid (CSF), or locally in the tumor cavity (Figure 1). Brain tumors are currently the most common solid tumor types undergoing clinical trial testing for CAR T cell efficacy and have shown early promise in the treatment of glioblastoma (GBM) (16). In this review, we focus on pediatric brain tumors as novel interventions are needed given the grim prognoses for many patients.

## Pediatric Brain Tumors

### Medulloblastoma

Medulloblastoma is the most common malignant brain tumor in children (10–20% of all pediatric brain tumors) with an incidence rate of 6.0 per million in patients 1–9 years old (17). Until recent years, medulloblastoma prognosis and classification was primarily stratified on a histological basis, as well as characteristics such as age and metastatic status (18). With increasing accessibility to advanced molecular genetic techniques, medulloblastoma has been further classified based on its distinct molecular subtypes (WNT, SHH, Group 3, and Group 4), shedding new light on potential therapeutic targets (19). These tumors develop in the cerebellar vermis and thus are exclusively in the posterior fossa (20).

The current standard of care for medulloblastoma remains surgical resection, craniospinal irradiation, and chemotherapy. The expected 5-year survival is 70–75% amongst children greater than 3-years-old (21–26). New risk stratification groups and survival outcomes have been proposed taking into account the molecular subtypes. After review of data from several cohorts of the 4 molecular subtypes, Ramaswamy et al. proposed a four group stratification scheme: low risk (>90% survival), standard risk (75–90% survival), high risk (50–75% survival) and very high risk (<50% survival). The WNT subtype and MYCN-amplified SHH subtype had the best and worst overall survival, respectively (27). The prognostic information provided by molecular subtyping can influence treatment specifically the need for adjuvant radiation given the increased risk of neurocognitive impairment in children (28).

#### Molecular Subtypes

The WNT subgroup of medulloblastoma, known for its excellent long-term prognosis compared to other subgroups, has mortality outcomes related more to complications of treatment or secondary neoplasms rather than tumor recurrence (19, 29). The classic WNT pathway defects in this subgroup occur through somatic deletions of CTNNB1 on chromosome 6 (encoding b-catenin), or monosomic deletions of chromosome 6, and thus have positive immunohistochemical staining for b-catenin (30). Additionally, germline mutations of WNT pathway inhibitor APC are associated with Turcot syndrome and associated medulloblastoma (31). Current studies in this subgroup aim to improve excellent survival outcomes while decreasing whole brain and spine radiation doses (NCT01878617, NCT02724579).

The SHH (sonic hedgehog) group is characterized by mutations resulting in activation of the SHH pathway. Germline mutations in SHH receptor PTCH (Gorlin syndrome) and SHH inhibitor SUFU predispose to medulloblastoma, especially infantile forms (32–36). This activation occurs primarily through somatic PTCH1/SMO/SUFU mutations, as well as amplifications of GLI1, GLI2, and MYCN (34, 37, 38). Tumors of this group occur in all ages and is the predominant subgroup in children <3 years of age and adults. Outcomes are good in young children with a less favorable outcome in older children and adults, especially with TP53 mutations (27, 39).

Group 3 tumors are characterized primarily by MYC amplification, as opposed to MYCN amplification characteristic of the SHH subgroup (40–42). Amplification of oncogene OTX2 can also be seen in group 3, as well as group 4 tumor (43–45). Furthermore, while the pathogenesis is not yet clear, these tumors frequently overexpress genes involved in retinal development and GABAergic pathways (40–42). Overall, these tumors frequently metastasize and thus have the worst outcome of all subgroups (19, 27).

Group 4 tumors remain the least understood of the subgroups, however, they make up >30% of all medulloblastomas (19). Isochromosome 17q is a common feature of group 4 tumors, occurring in 66% of tumors (41, 46). Also notable is the high incidence of cytogenetic loss of the X chromosome in 80% of females with group 4 medulloblastomas (40). These tumors frequently metastasize and can be high risk depending on their genetic features (27). Further research on the etiopathogenesis of medulloblastoma is ongoing.

#### Antigenic Targets

Receptor tyrosine-protein kinase ERBB2 (HER2) expression is most well-known for its role in a subset of breast cancers; however, HER2 expression is seen in approximately 40% of medulloblastomas (47). Given that ERBB2 protein is not detected in normal brain (48), this makes it an attractive target for CAR T cell therapy. Treatment with monoclonal antibodies targeting HER2 was ineffective in medulloblastoma likely due to lower expression profiles than in breast cancer and lack of HER2 gene amplification (49).

Early application with first generation CAR T cells (i.e., CAR T cells with an intracellular CD3 zeta domain and no co-stimulatory domain) in targeting HER2 showed promise by demonstrating effective targeting and regression of medulloblastomas in an orthotopic xenogenic mouse model (49). Efficacy of this study was likely limited by reliance on use of first generation CD3 constructs. Nellan et al. recently showed improved response and durable regression when using second generation CAR T cells with 4-1BB co-stimulation administered regionally to target HER2 in a preclinical xenograft model (50). These second generation CAR T constructs have shown improved persistence, increased T cell activation and decreased T cell exhaustion (51).

B7-H3 (CD276) overexpression has been found in a variety of human cancers, including lung adenocarcinoma, craniopharyngioma, neuroblastoma, medulloblastoma, glioma, ovarian cancer, pancreatic cancer, and acute myeloid leukemia. This pan-cancer antigen has absent or low in normal tissues making it an ideal CAR T cell target (52). In xenograft models of pediatric osteosarcoma, medulloblastoma, and Ewing sarcoma, B7-H3 CAR T cells demonstrated efficacy against tumors high surface target antigen density (53). Given the heterogeneity of cell surface antigen expression in brain tumors, such as glioblastoma (GBM), multivalent CAR T cells have been designed. These CAR T cells are capable of targeting multiple antigens simultaneously. Trivalent targets to EPHA2, HER2, and IL13Rα2 with CAR T cells has demonstrated efficacy in preclinical models of recurrent medulloblastoma and GBM (3, 54).

Targeting cancer testis antigens is of interest given their limited normal tissue expression on testicular germ cells and placental trophoblasts, thereby decreasing off target effects (55). The cancer testis antigen, PRAME, has been estimated to be expressed in up to 80% of medulloblastomas and could potentially serve as an immunotherapeutic target (47). Many cancer testis antigens remain MHC restricted immune targets making widespread application difficult due to the vide variance of MHC alleles across populations. However, Orlando et al. recently showed some success in orthotopic medulloblastoma models using CAR-T cells specific for the PRAME-derived peptide SLL (56). This peptide, which is presented in context of HLA-A^∗^02, is thought to be present in up to 48.4% in caucasians and 22.6% in black ethnic groups (57).

#### Tumor Microenvironment

The tumor microenvironment is comprised of tumor-infiltrating immune cells, fibroblasts, and endothelial cells, as well as a dynamic extra-cellular matrix which all can modulate tumor progression and the response to immunotherapy (58). The tumor microenvironment in solid tumors is typically immunosuppressive and impairs the efficacy of immunotherapy including CAR T cell therapy (59). Pediatric brain tumors are less immunosuppressive compared to their adult counterparts (60). The tumor microenvironment is still being characterized in medulloblastoma but there are differences in tumor infiltrating leukocytes between molecular subtypes. SHH group tumors are characterized by higher immune cell infiltration such as tumor associated macrophages (TAMs) and increased expression of inflammation-related genes compared to group 3 and 4 tumors (61, 62). Interestingly, Pham et al. measured immunosuppressive subsets of CTLA-4 or PD-1-expressing T cells in murine Group 3 tumors and found they contained higher percentages of PD-1+ and CD8+ T cells (62). In xenograft models, a significant survival benefit was only present in group 3 medulloblastoma subtypes treated with anti-PD-1 alone or in combination with anti-CTLA-4 (62, 63). The differential response to anti-PD-1 blockade observed in group 3 medulloblastoma suggests that the PD-1/PD-L1 environment is a key immunoregulatory pathway.

In previous small human cohort studies, there was no subgroup specific patterns of tumor infiltrating lymphocytes observed (64, 65). Murata et al. reported that 56% of cases had high expression of PD-L1 and was actually associated with low CD8 + T cell infiltration and poor prognosis (66). However, two other cohort studies reported no significant expression of PD-L1 in any of the studied cases of medulloblastomas (64, 65). A recent, more comprehensive study, by Bockmayr et al. which included 763 medulloblastoma cases did find significant differences in tumor microenvironments between subgroups using gene expression data analysis (67).

### Pediatric Ependymoma

Ependymomas comprise 5.2% of pediatric CNS tumors making these tumors the third most common in the pediatric population (68, 69). These tumors arise from cells along the lining of the cerebral ventricles or the spinal cord central canal. For the purposes of this review we will only discuss intracranial ependymomas. Ependymomas are classified by the World Health Organization (WHO) as grades I, II, and III based on their grade of anaplasia (70). Standard therapy includes aggressive gross total resection (GTR) is combination with radiotherapy. Merchant et al. showed that conformational radiation therapy (CRT) significantly improves survival in children and is similar across pedatric age groups including patients less than 3 years of age (71, 72). Aggressive GTR is critical in ependymoma patients to prevent recurrence and can sometimes be difficult given local infiltration. Recurrent disease can be difficult to manage and overall survival at 5 years is as low as 37% for recurrent tumors (72, 73). While the utility of radiotherapy has clearly been shown in treating ependymomas in children, the benefit with upfront chemotherapy is less understood. However, a recently closed trial exploring this will soon be reporting results (COG trial ACNS0831).

#### Molecular Supbtypes

With advances in molecular genetics, we now know that ependymomas have four subtypes, two in the posterior fossa and two in the supratentorial space. Posterior fossa ependymomas (PFE) have 2 distinct groups, PFE group A (PFA) and group B (PFB), each of which have distinct demographics, epigenetics, and outcomes (74, 75). PFA tumors are typically only found in infants, while PFB occurs equally in adults and adolesents (76). Patients with PFA have increased risk of recurrence and worse overall survival (75).

Supratentorial ependymoma are divided into two subtypes based on mutational drivers namely, C11orf95-RELA (RELA) fusions and YAP1 (YAP) fusions. These two supratentorial subtypes are genetically and clinically distinct with RELA patients having a poorer prognosis than those with YAP (76).

#### Antigenic Targets

Studies have demonstrated increased expression of EphA2, IL-13Rα2, HER2 and Survivin in ependymomas (77, 78). Therefore, these antigens may be potentially effective targets in CAR T cell mediated therapy clinically. CAR-T cells with trivalent targets to EPHA2, HER2 and IL13Rα2 did show efficacy in xenograft models of ependymomas (3). While ependymomas may hold favorable outcomes with traditional therapy in some pediatric patients, recurrence is often fatal and further work in novel treatments and immunotherapy is needed.

#### Tumor Microenvironment

Similar to medulloblastoma, tumor microenvironment characteristics directly correlate with molecular subgroup. PFA tumors have enrichment in inflammatory response genes in comparison to PFB tumors (79). IL6/STAT3 pathway activation and crosstalk between cancer cells and myeloid cells is a potential mechanism underlying the PFA tumor phenotype. This pathway could potentially be used as a therapeutic target (80). RELA ependymomas have higher PD-L1 gene expression in comparison to other subtypes indicating a potential increased immunosuppression in this group (81). Further immune phenotyping of ependymomas with microarrays demonstrated that myeloid cells in the microenvironment have a more M1 or pro-inflammatory phenotype (82). Charactization of primary and matched recurrent ependymoms demonstrate that some tumors did change molecular subtype and inflammatory profiles upon recurrence (83). This indicates that the microenvironmnet can change during a patient’s treatment course, a critical consideration when choosing a therapy such as CAR T cells.

### Atypical Teratoid/Rhabdoid Tumors

Atypical teratoid rhabdoid tumors (ATRTs) are aggressive embryonal CNS tumors that occur in 0.66 per 100,000 children. These tumors usually occur in children less than 4 years old and can occur in the posterior fossa or the supratentorium. The majority of tumors in children younger than 1 year occurred infratentorially. ATRT dianosis portends a grave diagnosis with a median survival less than 1 year after diagnosis for most patients (84–86). No standard treatment exists for this tumor but surgical resection is indicated. Given the young age of the patient at presentation, radiation treatment can lead to severe neurocognitive deficits. Despite newer therapies such as intensified multimodal therapy with whole craniospinal irradiation (87, 88) or high-dose chemotherapy with stem-cell rescue (89, 90) showing improved survival, they come with significant treatment-related morbidity and mortality (91). With the further classification of ATRTs using integrated (epi)-genomic analysis, use of targeted/biological agents may show further promise for some subtypes of ATRTs (92, 93).

#### Molecular Subtypes

The hallmark of ATRTs is biallelic mutations of SMARCB1 (INI1/hSNF5/BAF47), or rarely SMARCA4, both of which are involved in the SWItch/Sucrose Non-fermentable chromatin remodeling complex (94). Loss of one copy of the entire chromosome 22 or a deletion or translocation specifically involving chromosome band 22q11.2 has been described (95). Recent epigenetic studies have further subdivided ATRT into three methylation subgroups, ATRT-SHH, ATRT-TYR, ATRT-MYC, each of which has characteristic locations and methylation patterns (96).

#### Antigenic Targets

To date, no definitive ATRT neoantigens have been described, however, preclinical data with cancer stem cells and tyrosine kinase inhibitors in ATRTs may highlight potential antigens for CAR-T targeting and trafficking. Cancer stem cells (CSCs) are subpopulations of tumors cells thought to be associated with treatment failure, tumor recurrence, and metastasis of many cancers primarily due to their quiescence, migratory ability, resistance to drug therapy, and immune evasion (97). Use of patient-derived xenografts (PDXs) to identify and isolate human (CSCs)/tumor-initiating cells (TICs) demonstrated distinct gene signatures associated with CSCs/TICs in malignant rhabdoid tumors (98). Two of these overexpressed genes, CXCL5/CXCL6, may have potential for adjuvant CAR-T therapeutic targeting.

CXCL5/CXCL6 are both chemokine proteins thought to play a role not only in chemotaxis, but angiogenesis and tumorigenesis in many cancer types (99–101). CXCL5 has recently come to the forefront in research for its involvement in proliferation, migration, invasion, and immune evasion in multiple cancer types. Integrated proteomics data suggests that these chemokines are not normally expressed in nervous system tissues, a key factor in trafficking and minimizing off-tumor toxicity for ATRT therapy (102, 103). While these ligand are not viable targets, their receptors could potentially be used as targets for adoptive cell therapy.

Another potential antigenic target for CAR-T therapy is PTK7, a RTK family member, single-pass transmembrane receptor that lies at the signaling crossroads of WNT, VEGF, and stem cell function (104). Recent studies have shown a significant increase in PTK7 RNA expression levels in ATRT tumors, with subsequent repression of proliferation and viability of ATRT cells by vatalanib, which target multiple tyrosine kinases, and siRNA-mediated PTK7 knockdown, respectively (105). Preclinical data with antigen-drug conjugate, anti-PTK7 (PF-06647020), in breast, ovarian, and non-small cell lung cancer, shows that PTK7 targeting of CSCs and the tumor microenvironment induces direct and indirect anti-tumor effects, by reducing tumor initiating cells and inducing sustained tumor regression (106).

There are many ongoing and completed clinical trials for patients with ATRTs involving combination therapies, biological agents, and enzyme inhibitors, as well as molecular genetics studies. Much work is needed in preclinical studies to further delineate neoantigens for CAR-T cell targeting in this aggressive pediatric brain tumor.

#### Tumor Microenvironment

ATRT are rare tumors and therefore studies on characterizing the tumor microenvironment are limited. There is critical crosstalk between mesenchymal stromal cells and ATRT cancer cells in the microenvironment that leads to the promotion of cancer cell migration (107). The tumor immune microenvironment in ATRTs appears to have some unique features. Tumor immune phenotyping demontrated that ATRTs have significantly increased CD8 T cells in comparison to GBMs (108). Similar to other pediatric tumors, the immune infiltrate composition corresponds with the molecular subtype. Macrophages predominantly infiltrate the ATRT-SHH and ATRT-MYC subtypes and correlate with poor prognosis and treatment resistance (109). These data again indicate that therapeutic strategies must take into account the subtype classification and its respective immune microenvironment properties in pediatric brain tumors.

### Pediatric High Grade Gliomas

Pediatric High-Grade Gliomas (pHGGs) represent less than 20% of tumors in this population, and are comprised primarily of anaplastic astrocytomas (AA, WHO grade III), glioblastoma (GBM, WHO grade IV), and the newly defined Diffuse Midline Glioma (DMG, WHO grade IV) (110–114). Pediatric non-brainstem/midline glioblastomas (pNMGs) are often cortical and thus treated with aggressive combinations of surgery, chemotherapy, and radiation due to poor prognosis (114). Even with gross total resection and chemotherapy, 3-year event free survival is reported to only be 11% in children with high-grade pNMG (115).

The 2016 WHO classification of CNS tumors introduced a new classification known as H3 K27M-mutant diffuse midline gliomas (DMG), which refers to the specific lysine-to-methionine substitution at position 27 in histone 3 (70, 112, 116). These tumors, as their name suggests, arise in midline structures such as the thalamus, brainstem, and spinal cord. They carry a dismal prognosis due to the eloquent location of these tumors making surgery not feasible. The DMG classification thus includes tumors previously referred to as diffuse intrinsic pontine glioma (DIPG).

No standard therapy currently exists for DMG. Treatments include radiation therapy (117, 118), targeted chemotherapy (119, 120), and several types of adjuvant therapy capable of targeting cellular mechanisms of tumor proliferation, cell cycle progression, angiogenesis and DNA repair (121). Due to the difficult anatomical location of these tumors, surgical resection and even biopsies for tissue characterization, remains uncommon.

#### Molecular Subtypes

In contrast to adult gliomas, pediatric non-brainstem/midline glioblastomas (pNMGs) in children more often have TP53 mutations, as opposed to EGFR or PTEN alterations (114, 122). Glioblastomas in children however, almost exclusively arise de novo compared to the secondary development in IDH-mutants from low grade gliomas in adults (122). NMGs in children are also associated with genetics syndromes such as Turcot syndrome, Li-Fraumeni syndrome, and Neurofibromatosis type 1, all of which have their own known associated mutations in the context of tumor development.

In pediatric high-grade gliomas, approximately 40% are associated with TP53 mutations, and low p53 expression correlates with improved 5-year progression-free survival (114, 122). Overall, compared to adult high-grade gliomas, pNMGs are less likely to have EGFR gene amplification and less likely to have mutations within the PTEN tumor suppresor gene, although correlations with prognosis and clincial significance are limited (123–125).

Due to the mortality of DMG tumors and difficulty of surgical resection, many clinical trials have attempted to target genetic mutations in the tumor. Early trials showed little success as many targeted the genetic mutations known to adult glioblastomas (126). As more genetic studies have been completed, there are clear differences in gene expression that distinguish DMG from both adult glial tumors and supratentorial pediatric gliomas (127, 128). The first of these differences to be uncovered was that nearly 80% of DIPG tumors contained frequent histone 3 mutations (K27M-H3.3 or K27M-H3.1) (111, 112, 116). Later studies further subcatagorized DIPGs into three molecularly distinct subgroups (H3-K27M, Silent, and MYCN) (129).

#### Antigenic Targets

Antigen targeting potential in high grade gliomas is currently the most robust due to translation of research from adult high-grade glioma counterparts. The main antigen targets in adult gliomas inlude: IL13Rα*2*, EphA2, EGFRvIII, and HER2 (1). CD70, a member of the tumor necrosis factor receptor family, and podoplanin (PDPN), a type I transmembrane mucin-like glycoprotein, have also been recently identified as a CAR T cell target in glioma (130, 131). Table 1 summarizes antigen targets for pediatric brain tumors including pHGG. While pediatric clinical trials are in progress that include these antigens, recruitment is more limited due to the difference in antigen expression seen between adult and pediatric high-grade gliomas. However, some activity has been seen in the pediatric population. A prospective trial including 17 adult and adolescent patients with relapsed or refractory glioblastoma included an adolescent patient with an objective response after systemically administered HER2 CAR T cells (132).

**TABLE 1:** Primary antigens targeted in CAR-T therapy for pediatric brain tumors.

**Antigen**	**Tumor expression**	**Function**	**References**
EGFRVIII	Glioblastoma, Ependymoma, Medulloblastoma	Brain development and is associated with tumor development and progression	(186, 187)
HER-2	Glioblastoma, Ependymoma, Medulloblastoma, metastatic tumors	Cell cycle homeostasis	(49, 54, 132)
B7-H3	Diffuse Midline gliomas, medulloblastoma, and most pediatric solid tumors	Potent immune inhibitory functions	(53, 136)
GD2	Primarily Diffuse Midline Gliomas, neuroblastoma, melanoma	Attachment of tumor cells to extracellular matrix proteins	(138, 188)
IL13RA 2	High-grade glioma, Ependymoma, Medulloblastoma	Apoptosis escape mechanism	(54, 189, 190)
EPHA2	Gliomas, Ependymoma, Medulloblastoma	Enhances tumorigenesis, tumor cell migration and invasion, angiogenesis, and metastasis	(54, 191, 192)
SURVIVIN	Ependymoma, glioma	Apoptosis inhibitor protein	(142)
PRAME	Medulloblastoma	Inhibits retinoic acid signaling	(47)
CD 70	Glioma	Tumor necrosis factor receptor family	(130)
PDPN	Carcinoma, sarcoma, seminoma, brain tumors	Transmembrane glycoprotein	(151)

In pediatric diffuse midline gliomas, both B7-H3 and GD2 specific CAR-T cells, which are highly expressed in DMGs, are also currently targets in clinical trials recruiting pediatric patients (NCT04185038, NCT0409979). B7-H3 is a transmembrane protein that belongs to the B7 immune co-stimulatory and co-inhibitory family, has potent immune inhibititory function (133, 134). Studies have shown that it downregulates T-cell activation and INFg, and correlates with fewer tumor-infiltrating lymphocytes, faster cancer progression, and poor clinical outcome in multiple malignancies (135, 136). In one study, it was demonstrated that 100% of DIPG specimens were B7-H3 immunoreactive, and mRNA expression was significantly higher in DIPG samples vs juvenile pilocytic astrocytoma or normal brain (137). Majzner et al. demonstrated that B7-H3 CAR T cells in mice significantly prolonged survival in both medulloblastoma and DIPG xenografts through production of IFNg, TNFa, and IL2 (53).

A recently published study demonstrated the potent anti-tumorgenic capabilities of CAR-T cells targeted to pediatric diffuse midline gliomas (138). In these tumors, the presence of the H3-K27M mutation correlated with high levels of GD2 diganglioside expression. Many immunotherapies targeting GD2 expression are being investigated in both preclinical and clinical trials for a variety of neurologic conditions including neuroblastoma (139–142). To date, CAR-T cell therapies targeting GD2 expression in neuroblastoma have been well tolerated in many of these clinical trials (139–141).

Due to the known heterogenicity of high-grade gliomas and potential for antigen escape, multi-valent immunotherapy could prove to be a superior approach in treating recurrent gliomas in both adult and pediatric populations. Pollack et al. showed feasibility, tolerability, and some evidence of efficacy in using a trivalent peptide vaccine targeting EphA2, IL13Rα2, and survivin in pediatric malignant gliomas (143). Similar preliminary work has also shown potential efficacy for pediatric and adult ependymomas (77). Early CAR-T studies showed the benefit of targeting multiple antigens and decreasing tumor antigen escape (144). Bielamowicz et al. has also demonstrated that trivalent CAR-T cells targeting HER2, IL13Rα2, and EphA2 demonstrated superior antitumor activity in GBM patient derived xenografts compared to patient-specific single valent and bivalent models (54). A multivalent is particularly useful in HGG given the tumor heterogeneity of antigen expression which can change over time following CAR T cell therapy (145).

#### Challenges in the Microenvironment

pHGGs have been shown to differ from their adult counterparts through the presence of varying somatic histone modifications (110–112). Differential genetic expression between adult and pediatric gliomas result in proteomes with varied biomarker expression with subsequent influences on the tumor microenvironment. Although still controversial, it is thought the pHGGs may arise from neural precursor cells of the oligodendroglial lineage (113, 146, 147). These types of tumors have been shown to possess distinct discrete patterns of spatial and temporal development that correspond with periods of developmental myelination within the pediatric and adolescent brain (110, 113, 148, 149).

In adult gliomas, the heterogenity of both antigen expression and the tumor microenvironment proves to be a main hurdle in using adoptive cell therapies like CAR-T cells (1, 150). Genetic sequencing of spatially different regions of GBMs has shed light on these regional niches within the tumor containing multiple cell types and functions (151, 152). As with adult glioma tumors, infiltrating immune cells play an important role in the makeup of the pediatric tumor microenvironment. Although the knowledge regarding the functional roles of such cells in pediatric gliomas remain incomplete, in mouse models of low-grade pediatric gliomas, tumor associated macrophages have been implicated in promotion of tumor cell proliferation (153, 154). Similarly, in adult gliomas, the amount of infiltrating TAMs that are CD68 + increases with increasing tumor grade (155). This signifies a correlation between immune cell presence within the tumor microenvironment and tumor malignancy and growth.

While little is known about the differences between tumor immune microenvironments in pedatric versus adult gliomas, some studies have shown that pDMGs show unique properties (156). In contrast to pNMGs, pDMGs do not have increased T cell infiltration compared to adjacent non-tumor infiltrated brain and appears to not have an inflammatory cytokine profile (157). Sequencing of macrophages from tissue samples in both pDMGs and adult GBM have shown that both express genetic changes related to extracellular matrix remodeling and angiogenesis. However, pDMG-associated macrophages express less inflammatory factors than adult GBM macrophages (158). Thus, the lack of T-cell invasion and overall low inflammatory environment could potentially make pDMGs excellent candidates for CAR-T cells as the microenvironment is less immunosuppressive relative to adult tumor counterparts (158, 159).

## Treatment Toxicity and Risks

CAR T cell therapy can lead to treatment associated toxicity. Cytokine release syndrome (CRS) is one of the most common and severe toxicities associated with CAR T cell therapy results from systemic immune activation. While elevation of inflammatory cytokines is expected, and often produces mild flu-like symptoms, multiorgan system failure and death has been reported from CRS (160). The most elevated cytokines typically seen are IL-10, IL-6, and IFN-γ. CRS can be managed with the use of corticosteroids and interleukin-6 blockade (161, 162). Corticosteroids can have a negative impact on T-cell proliferation and therefore clinicians may consider other forms of CRS management. Out of the 3 cytokines involved, regulation of IL-6 should produce both desired control of CRS without downregulation of desired immune function (163). IL-6 blockade via tocilizumab has shown dramatic reversal of severe CRS in patients treated with CART-19, and thus is actively being studied in other therapies (164, 165). Another method managing severe CRS is engineering suicide genes into the CAR-T cells. With a suicide gene/switch in place, administration of an antibody or small molecule can induce apoptosis of CAR-T cells to prevent further immune stimulation and reverse CRS (166–168).

Despite the availability of multiple treatment modalities, CRS can still be deadly and needs to be recognized early (169). Neurotoxicity is another complication which has recently been termed immune effector cell-associated neurotoxicity syndrome (ICANS). ICANS is the second most common adverse event following CAR T-cell infusion. ICANS has a wide spectrum of symptoms and can range from mild confusion to being in a comatose state (170). Similar to CRS, ICANS can be deadly and therefore any neurological symptom occurring after CAR T cell infusion must be considered potential ICANS until proven otherwise. In a meta-analysis of CAR T cell trials for cancer, 55.3 and 37.2% of all patients experienced CRS and neurotoxicity, respectively (171).

Intrathecal or locoregional infusion of CAR T cells for brain tumor therapy may mitigate these toxicities experienced following intravenous administration (Figure 1). Intrathecal therapy is a passive form of delivery and normal CSF flow is required for adequate dissemination throughout the CNS. Furthermore, this modality may not penetrate bulky disease sites (172). These are clinical considerations to account for when choosing a delivery method. One other particular concern in regards to pediatric brain tumors is the prediliction for tumors in the posterior fossa or along the ventricular pathway. In preclinical models, expected treatment related inflammation following CAR T cell therapy can cause brain swelling which can lead to hydrocephalus (137). Hydrocephalus can be morbid and require urgent CSF diversion. These risks may occur more frequently as the clinical experience expands in pediatric brain tumors.

## Discussion and Future Directions

Overall, current clinical trials using CAR-T cells for pediatric brain tumors are in early stages (Table 2). The antigenic targets of these trials have been discussed in previous sections and are summarized in Table 1. Five clinical trials are currently recruiting pediatric patients, however, results will likely be slow to report due to the combined rarity and mortality of these tumors. However, CAR-T therapy in xenograft models continues to provide hope in reaching long-term disease free survival.

**TABLE 2:** Ongoing clinical trials using chimeric antigen-receptor T-cells (CAR-T) for pediatric brain tumors.

**NCT#**	**Phase**	**Study Name**	**Responsible party**	**Target**	**Additional agents/therapies**	**Delivery**	**Disease**
03638167	Phase I	EGFR806-specific CAR T Cell Locoregional Immunotherapy for EGFR-positive Recurrent or Refractory Pediatric Central Nervous System Tumors	Seattle Children’s Hospital	EGFR		IT, IC	Pediatric Glioma, Ependymoma, Medulloblastoma, Germ Cell Tumor, ATRT, PNET, Choroid Plexus Carcinoma, Pineoblastoma
03500991	Phase I	HER2-Specific CAR T Cell Locoregional Immunotherapy for HER2 Positive Recurrent/Refractory Pediatric Central Nervous System Tumors	Seattle Children’s Hospital	HER2		IT, IC	Pediatric Glioma, Ependymoma, Medulloblastoma, Germ Cell Tumor, ATRT, PNET, Choroid Plexus Carcinoma, Pineoblastoma
04185038	Phase I	B7-H3-Specific CAR T Cell Locoregional Immunotherapy for Diffuse Intrinsic Pontine Glioma/Diffuse Midline Glioma and Recurrent or Refractory Pediatric Central Nervous System Tumors	Seattle Children’s Hospital	B7-H3		IT, IC	DIPG/DMG, Ependymoma, Medulloblastoma, Childhood Germ Cell Tumor, ATRT, PNET, Choroid Plexus Carcinoma, Pineoblastoma, Childhood Glioma
02442297	Phase I	Intracranial Injection of T Cells Expressing HER2-specific Chimeric Antigen Receptors (CAR) in Subjects With HER2-Positive Tumors of the Central Nervous System (iCAR)	Texas Children’s Hospital	HER2		IT, IC	Brain Tumor, Recurrent Brain Tumor, Refractory Excludes DIPG/DMG
04099797	Phase I	Autologous T Lymphocytes Expressing GD2-specific Chimeric Antigen and Constitutively Active IL-7 Receptors for the Treatment of Patients With GD2-expressing Brain Tumors (GAIL-B)	Texas Children’s Hospital	GD2	Lymphodepletion chemotherapy; Cyclophosphamide, Fludarabine	IV	Diffuse Intrinsic Pontine Glioma (DMG), High Grade Glioma

*Table data accrued July 19, 2020 from ClinicalTrials.gov.*

The first in-human studies of CAR-T cells for GBM was administered through intravenous delivery. O’Rourke et al. demonstrated that with while all patients had detectable transient expansion of EGFRvIII CAR-T cell in peripheral blood, not all patients had significant signs of trafficking and intra-tumor effects (173). Ahmed et al. showed a dramatic response in an adolescent patient, and stable disease in 7 others after HER2 CAR-T infusion (132). Brown et al. was among the first to show regression of GBM in a patient after intratumor and intraventricular infusion of IL13Rα2 CAR-T cells (145). Route of administration that optimizes efficacy will be a main factor to delineate in future trials.

Assessing response to immunotherapy remains another hurdle to overcome in the context of CNS cancer. The standard of care in monitoring tumor progression and response is magnetic resonance imaging, but, pseudo-progression after immunotherapy can make interpretation of imaging difficult. Pseudo-progression and immune related edema is an expected result of targeting and stimulating the immune system, and more advanced imaging analysis stratagies are underway in helping to differentiate between true progression of disease (174–176). Despite these concerns, multiple clinical trials have shown that patients still achieve meaningful clinical response despite early pseudo-progression findings (174, 177–181).

Currently, trials are targeting cell surface antigens on cancer cells but scFv targeting epitope/HLA complex makes it possible for intracellular proteins to be candidate targets (182). This technology will be a significant game changer allowing for clinicians to target intracellular oncogenic pathways. Besides targeting cancer cells, considerations of how to target the microenvironment to enhance anti-tumor activity is underway (183). In pediatric brain tumors, further work is needed to delinate the tumor microenvironment taking into account subtype variability. With the advent of immunomodulatory agents such as checkpoint inhibition, multimodal immunotherapy can also be considered for patients (184). Lastly, the application of CAR engineering to other immune effector cells such as NK cells opens another avenue of potential innovation (185).

## Conclusion

There has been critical advancements in the last decade that have allowed for the development of CAR T cells for brain tumors. The prognosis for many pediatric brain tumors remains dismal and there is promise in CAR T cell therapy. Continued preclinical research is needed to address our current gaps such as identifying the best mode of delivery, defining the microenvironment, developing new targets and increasing efficacy. Despite these challenges the future of CAR T cells for pediatric brain tumor management is bright.

## Author Contributions

JP: methodology, data curation, and writing – original draft, review, and editing. JH: writing – original draft. RB: figure construction. KB: supervision and writing – review and editing. AR: conceptualization, resources, supervision, and writing – review and editing. All authors contributed to the article and approved the submitted version.

## Conflict of Interest

The authors declare that the research was conducted in the absence of any commercial or financial relationships that could be construed as a potential conflict of interest. The handling Editor declared a past co-authorship with one of the authors KB.

## References

[1] Rodriguez A Brown C Badie B. Chimeric antigen receptor T-cell therapy for glioblastoma. *Transl Res.* (2017) 187:93–102. 10.1016/j.trsl.2017.07.003 28755873

[2] Mirzaei HR Rodriguez A Shepphird J Brown CE Badie B. Chimeric antigen receptors T cell therapy in solid tumor: challenges and clinical applications. *Front Immunol.* (2017) 8:1850. 10.3389/fimmu.2017.01850 29312333 PMC5744011

[3] Donovan LK Delaidelli A Joseph SK Bielamowicz K Fousek K Holgado BL Locoregional delivery of CAR T cells to the cerebrospinal fluid for treatment of metastatic medulloblastoma and ependymoma. *Nat Med.* (2020) 26:720–31. 10.1038/s41591-020-0827-2 32341580 PMC8815773

[4] Withrow DR De Gonzalez AB Lam CJK Warren KE Shiels MS. Trends in pediatric central nervous system tumor incidence in the United States, 1998–2013. *Cancer Epidemiol Biomarkers Prev.* (2019) 28:522–30. 10.1158/1055-9965.EPI-18-0784 30464022 PMC6401271

[5] Gross G Waks T Eshhar Z. Expression of immunoglobulin-T-cell receptor chimeric molecules as functional receptors with antibody-type specificity. *Proc Natl Acad Sci USA.* (1989) 86:10024–8. 10.1073/pnas.86.24.10024 2513569 PMC298636

[6] Eshhar Z Waks T Gross G Schindler DG. Specific activation and targeting of cytotoxic lymphocytes through chimeric single chains consisting of antibody-binding domains and the gamma or zeta subunits of the immunoglobulin and T-cell receptors. *Proc Natl Acad Sci USA.* (1993) 90:720–4. 10.1073/pnas.90.2.720 8421711 PMC45737

[7] Gaudino SJ Kumar P. Cross-talk between antigen presenting cells and T cells impacts intestinal homeostasis, bacterial infections, and tumorigenesis. *Front Immunol.* (2019) 10:360. 10.3389/fimmu.2019.00360 30894857 PMC6414782

[8] Finney HM Lawson AD Bebbington CR Weir AN. Chimeric receptors providing both primary and costimulatory signaling in T cells from a single gene product. *J Immunol.* (1998) 161:2791–7. 9743337

[9] Tammana S Huang X Wong M Milone MC Ma L Levine BL 4-1BB and CD28 signaling plays a synergistic role in redirecting umbilical cord blood T Cells against B-cell malignancies. *Hum Gene Ther.* (2010) 21:75–86. 10.1089/hum.2009.122 19719389 PMC2861957

[10] van der Stegen SJC Hamieh M Sadelain M. The pharmacology of second-generation chimeric antigen receptors. *Nat Rev Drug Discov.* (2015) 14:499–509. 10.1038/nrd4597 26129802 PMC6410718

[11] Fournier C Martin F Zitvogel L Kroemer G Galluzzi L Apetoh L. Trial Watch: adoptively transferred cells for anticancer immunotherapy. *Oncoimmunology.* (2017) 6:e1363139. 10.1080/2162402X.2017.1363139 29147628 PMC5674950

[12] Yang X Wang GX Feng ZJ. CAR T cell therapy for hematological malignancies. *Curr Med Sci.* (2019) 39:874–82. 10.1007/s11596-019-2118-z 31845217

[13] Ma S Li X Wang X Cheng L Li Z Zhang C Current progress in car-t cell therapy for solid tumors. *Int J Biol Sci.* (2019) 15:2548–60. 10.7150/ijbs.34213 31754328 PMC6854376

[14] Abbott NJ. Blood-brain barrier structure and function and the challenges for CNS drug delivery. *J Inherit Metab Dis.* (2013) 36:437–49. 10.1007/s10545-013-9608-0 23609350

[15] Arvanitis CD Ferraro GB Jain RK. The blood–brain barrier and blood–tumour barrier in brain tumours and metastases. *Nat Rev Cancer.* (2020) 20:26–41. 10.1038/s41568-019-0205-x 31601988 PMC8246629

[16] Akhavan D Alizadeh D Wang D Weist MR Shepphird JK Brown CE. CAR T cells for brain tumors: lessons learned and road ahead. *Immunol Rev.* (2019) 290:60–84. 10.1111/imr.12773 31355493 PMC6771592

[17] Smoll NR Drummond KJ. The incidence of medulloblastomas and primitive neurectodermal tumours in adults and children. *J Clin Neurosci.* (2012) 19:1541–4. 10.1016/j.jocn.2012.04.009 22981874

[18] Packer RJ Rood BR MacDonald TJ. Medulloblastoma: Present concepts of stratification into risk groups. *Pediatr Neurosurg.* (2003) 39:60–7. 10.1159/000071316 12845195

[19] Taylor MD Northcott PA Korshunov A Remke M Cho Y-J Clifford SC Molecular subgroups of medulloblastoma: the current consensus. *Acta Neuropathol.* (2012) 123:465–72. 10.1007/s00401-011-0922-z 22134537 PMC3306779

[20] Roussel MF Hatten ME. Cerebellum: development and medulloblastoma. *Curr Top Dev Biol.* (2011) 94:235–82. 10.1016/B978-0-12-380916-2.00008-5 21295689 PMC3213765

[21] Lu VM Pendleton C Brown DA Lakomkin N Cho S Miller KJ Shaping our understanding of medulloblastoma: a bibliometric analysis of the 100 most cited articles. *Clin Neurol Neurosurg.* (2020) 194:105895. 10.1016/j.clineuro.2020.105895 32497953

[22] Taylor RE Bailey CC Robinson KJ Weston CL Walker DA Ellison D Outcome for patients with metastatic (M2–3) medulloblastoma treated with SIOP/UKCCSG PNET-3 chemotherapy. *Eur J Cancer.* (2005) 41:727–34. 10.1016/j.ejca.2004.12.017 15763649

[23] Tarbell NJ Friedman H Polkinghorn WR Yock T Zhou T Chen Z High-risk medulloblastoma: a pediatric oncology group randomized trial of chemotherapy before or after radiation therapy (POG 9031). *J Clin Oncol.* (2013) 31:2936–41. 10.1200/JCO.2012.43.9984 23857975 PMC3732312

[24] Packer RJ Gajjar A Vezina G Rorke-Adams L Burger PC Robertson PL Phase III study of craniospinal radiation therapy followed by adjuvant chemotherapy for newly diagnosed average-risk medulloblastoma. *J Clin Oncol.* (2006) 24:4202–8. 10.1200/JCO.2006.06.4980 16943538

[25] Gandola L Massimino M Cefalo G Solero C Spreafico F Pecori E Hyperfractionated accelerated radiotherapy in the milan strategy for metastatic medulloblastoma. *J Clin Oncol.* (2009) 27:566–71. 10.1200/JCO.2008.18.4176 19075266

[26] Lannering B Rutkowski S Doz F Pizer B Gustafsson G Navajas A Hyperfractionated versus conventional radiotherapy followed by chemotherapy in standard-risk medulloblastoma: results from the randomized multicenter HIT-SIOP PNET 4 Trial. *J Clin Oncol.* (2012) 30:3187–93. 10.1200/JCO.2011.39.8719 22851561

[27] Ramaswamy V Remke M Bouffet E Bailey S Clifford SC Doz F Risk stratification of childhood medulloblastoma in the molecular era: the current consensus. *Acta Neuropathol.* (2016) 131:821–31. 10.1007/s00401-016-1569-6 27040285 PMC4867119

[28] Sayour EJ Mitchell DA. Immunotherapy for Pediatric Brain Tumors. *Brain Sci.* (2017) 7:137. 10.3390/brainsci7100137 29065490 PMC5664064

[29] Ellison DW Kocak M Dalton J Megahed H Lusher ME Ryan SL Definition of disease-risk stratification groups in childhood medulloblastoma using combined clinical, pathologic, and molecular variables. *J Clin Oncol.* (2011) 29:1400–7. 10.1200/JCO.2010.30.2810 20921458 PMC3525837

[30] Zurawel RH Chiappa SA Allen C Raffel C. Sporadic medulloblastomas contain oncogenic beta-catenin mutations. *Cancer Res.* (1998) 58:896–9. 9500446

[31] Hamilton SR Liu B Parsons RE Papadopoulos N Jen J Powell SM The molecular basis of turcot’s syndrome. *N Engl J Med.* (1995) 332:839–47. 10.1056/NEJM199503303321302 7661930

[32] Pastorino L Ghiorzo P Nasti S Battistuzzi L Cusano R Marzocchi C Identification of a SUFU germline mutation in a family with Gorlin syndrome. *Am J Med Genet A.* (2009) 149A:1539–43. 10.1002/ajmg.a.32944 19533801

[33] Bale SJ Falk RT Rogers GR. Patching together the genetics of Gorlin syndrome. *J Cutan Med Surg.* (1998) 3:31–4. 10.1177/120347549800300109 9677257

[34] Taylor MD Liu L Raffel C Hui C Mainprize TG Zhang X Mutations in SUFU predispose to medulloblastoma. *Nat Genet.* (2002) 31:306–10. 10.1038/ng916 12068298

[35] Slade I Murray A Hanks S Kumar A Walker L Hargrave D Heterogeneity of familial medulloblastoma and contribution of germline PTCH1 and SUFU mutations to sporadic medulloblastoma. *Fam Cancer.* (2011) 10:337–42. 10.1007/s10689-010-9411-0 21188540

[36] Brugières L Pierron G Chompret A Paillerets BB Di Rocco F Varlet P Incomplete penetrance of the predisposition to medulloblastoma associated with germ-line SUFU mutations. *J Med Genet.* (2010) 47:142–4. 10.1136/jmg.2009.067751 19833601

[37] Northcott PA Hielscher T Dubuc A Mack S Shih D Remke M Pediatric and adult sonic hedgehog medulloblastomas are clinically and molecularly distinct. *Acta Neuropathol.* (2011) 122:231–40. 10.1007/s00401-011-0846-7 21681522 PMC4538327

[38] Northcott PA Nakahara Y Wu X Feuk L Ellison DW Croul S Multiple recurrent genetic events converge on control of histone lysine methylation in medulloblastoma. *Nat Genet.* (2009) 41:465–72. 10.1038/ng.336 19270706 PMC4454371

[39] Zhukova N Ramaswamy V Remke M Pfaff E Shih DJH Martin DC Subgroup-specific prognostic implications of TP53 mutation in medulloblastoma. *J Clin Oncol.* (2013) 31:2927–35. 10.1200/JCO.2012.48.5052 23835706 PMC4878050

[40] Kool M Koster J Bunt J Hasselt NE Lakeman A van Sluis P Integrated genomics identifies five medulloblastoma subtypes with distinct genetic profiles, pathway signatures and clinicopathological features. *PLoS One.* (2008) 3:e3088. 10.1371/journal.pone.0003088 18769486 PMC2518524

[41] Northcott PA Korshunov A Witt H Hielscher T Eberhart CG Mack S Medulloblastoma comprises four distinct molecular variants. *J Clin Oncol.* (2011) 29:1408–14. 10.1200/JCO.2009.27.4324 20823417 PMC4874239

[42] Cho Y-J Tsherniak A Tamayo P Santagata S Ligon A Greulich H Integrative genomic analysis of medulloblastoma identifies a molecular subgroup that drives poor clinical outcome. *J Clin Oncol.* (2011) 29:1424–30. 10.1200/JCO.2010.28.5148 21098324 PMC3082983

[43] Di C Liao S Adamson DC Parrett TJ Broderick DK Shi Q Identification of OTX2 as a medulloblastoma oncogene whose product can be targeted by all-trans retinoic acid. *Cancer Res.* (2005) 65:919–24. 15705891

[44] De Haas T Oussoren E Grajkowska W Perek-Polnik M Popovic M Zadravec-Zaletel L OTX1 and OTX2 expression correlates with the clinicopathologic classification of medulloblastomas. *J Neuropathol Exp Neurol.* (2006) 65:176–86. 10.1097/01.jnen.0000199576.70923.8a 16462208

[45] Adamson DC Shi Q Wortham M Northcott PA Di C Duncan CG OTX2 is critical for the maintenance and progression of shh-independent medulloblastomas. *Cancer Res.* (2010) 70:181–91. 10.1158/0008-5472.CAN-09-2331 20028867 PMC2943736

[46] Kool M Korshunov A Remke M Jones DTW Schlanstein M Northcott PA Molecular subgroups of medulloblastoma: an international meta-analysis of transcriptome, genetic aberrations, and clinical data of WNT, SHH, Group 3, and Group 4 medulloblastomas. *Acta Neuropathol.* (2012) 123:473–84. 10.1007/s00401-012-0958-8 22358457 PMC3306778

[47] Orentas RJ Lee DW Mackall C. Immunotherapy targets in pediatric cancer. *Front Oncol.* (2012) 2:3. 10.3389/fonc.2012.00003 22645714 PMC3355840

[48] Uhlén M Fagerberg L Hallström BM Lindskog C Oksvold P Mardinoglu A Proteomics. Tissue-based map of the human proteome. *Science.* (2015) 347:1260419. 10.1126/science.1260419 25613900

[49] Ahmed N Ratnayake M Savoldo B Perlaky L Dotti G Wels WS Regression of experimental medulloblastoma following transfer of HER2-specific T cells. *Cancer Res.* (2007) 67:5957–64. 10.1158/0008-5472.CAN-06-4309 17575166

[50] Nellan A Rota C Majzner R Lester-McCully CM Griesinger AM Mulcahy Levy JM Durable regression of Medulloblastoma after regional and intravenous delivery of anti-HER2 chimeric antigen receptor T cells. *J Immunother Cancer.* (2018) 6:30. 10.1186/s40425-018-0340-z 29712574 PMC5925833

[51] Long AH Haso WM Shern JF Wanhainen KM Murgai M Ingaramo M 4-1BB costimulation ameliorates T cell exhaustion induced by tonic signaling of chimeric antigen receptors. *Nat Med.* (2015) 21:581–90. 10.1038/nm.3838 25939063 PMC4458184

[52] Zhang Z Jiang C Liu Z Yang M Tang X Wang Y B7-H3-targeted CAR-T cells exhibit potent antitumor effects on hematologic and solid tumors. *Mol Ther Oncolytics.* (2020) 17:180–9. 10.1016/j.omto.2020.03.019 32346608 PMC7178328

[53] Majzner RG Theruvath JL Nellan A Heitzeneder S Cui Y Mount CW CAR T cells targeting B7-H3, a Pan-cancer antigen, demonstrate potent preclinical activity against pediatric solid tumors and brain tumors. *Clin Cancer Res.* (2019) 25:2560–74. 10.1158/1078-0432.CCR-18-0432 30655315 PMC8456711

[54] Bielamowicz K Fousek K Byrd TT Samaha H Mukherjee M Aware N Trivalent CAR T cells overcome interpatient antigenic variability in glioblastoma. *Neuro Oncol.* (2018) 20:506–18. 10.1093/neuonc/nox182 29016929 PMC5909636

[55] Fratta E Coral S Covre A Parisi G Colizzi F Danielli R The biology of cancer testis antigens: putative function, regulation and therapeutic potential. *Mol Oncol.* (2011) 5:164–82. 10.1016/j.molonc.2011.02.001 21376678 PMC5528287

[56] Orlando D Miele E De Angelis B Guercio M Boffa I Sinibaldi M Adoptive immunotherapy using PRAME-specific T cells in medulloblastoma. *Cancer Res.* (2018) 78:canres.3140.2017. 10.1158/0008-5472.CAN-17-3140 29615432

[57] González-Galarza FF Takeshita LYC Santos EJM Kempson F Maia MHT Silva ALS Allele frequency net 2015 update: new features for HLA epitopes, KIR and disease and HLA adverse drug reaction associations. *Nucleic Acids Res.* (2015) 43:D784–8. 10.1093/nar/gku1166 25414323 PMC4383964

[58] Riaz N Havel JJ Makarov V Horak CE Weinhold N Chan TA. Tumor and microenvironment evolution during immunotherapy with nivolumab. *Cell.* (2017) 171:934–49.e16. 10.1016/j.cell.2017.09.028 29033130 PMC5685550

[59] Cheng J Zhao L Zhang Y Qin Y Guan Y Zhang T Understanding the mechanisms of resistance to CAR T-cell therapy in malignancies. *Front Oncol.* (2019) 9:1237. 10.3389/fonc.2019.01237 31824840 PMC6882288

[60] Haberthur K Brennan K Hoglund V Balcaitis S Chinn H Davis A NKG2D ligand expression in pediatric brain tumors. *Cancer Biol Ther.* (2016) 17:1253–65. 10.1080/15384047.2016.1250047 27834580 PMC5199167

[61] Margol AS Robison NJ Gnanachandran J Hung LT Kennedy RJ Vali M Tumor-associated macrophages in SHH subgroup of medulloblastomas. *Clin Cancer Res.* (2015) 21:1457–65. 10.1158/1078-0432.CCR-14-1144 25344580 PMC7654723

[62] Pham CD Flores C Yang C Pinheiro EM Yearley JH Sayour EJ Differential immune microenvironments and response to immune checkpoint blockade among molecular subtypes of murine medulloblastoma. *Clin Cancer Res.* (2016) 22:582–95. 10.1158/1078-0432.CCR-15-0713 26405194 PMC4922139

[63] Pham CD Mitchell DA. Know your neighbors: Different tumor microenvironments have implications in immunotherapeutic targeting strategies across MB subgroups. *Oncoimmunology.* (2016) 5:e1144002. 10.1080/2162402X.2016.1144002 27999733 PMC5139647

[64] Vermeulen JF Van Hecke W Adriaansen EJM Jansen MK Bouma RG Villacorta Hidalgo J Prognostic relevance of tumor-infiltrating lymphocytes and immune checkpoints in pediatric medulloblastoma. *Oncoimmunology.* (2018) 7:e1398877. 10.1080/2162402X.2017.1398877 29399402 PMC5790383

[65] Majzner RG Simon JS Grosso JF Martinez D Pawel BR Santi M Assessment of programmed death-ligand 1 expression and tumor-associated immune cells in pediatric cancer tissues. *Cancer.* (2017) 123:3807–15. 10.1002/cncr.30724 28608950

[66] Murata D Mineharu Y Arakawa Y Liu B Tanji M Yamaguchi M High programmed cell death 1 ligand–1 expression: association with CD8+ T-cell infiltration and poor prognosis in human medulloblastoma. *J Neurosurg.* (2018) 128:710–6. 10.3171/2016.11.JNS16991 28474991

[67] Bockmayr M Mohme M Klauschen F Winkler B Budczies J Rutkowski S Subgroup-specific immune and stromal microenvironment in medulloblastoma. *Oncoimmunology.* (2018) 7:e1462430. 10.1080/2162402X.2018.1462430 30228931 PMC6140816

[68] Korshunov A Witt H Hielscher T Benner A Remke M Ryzhova M Molecular staging of intracranial ependymoma in children and adults. *J Clin Oncol.* (2010) 28:3182–90. 10.1200/JCO.2009.27.3359 20516456

[69] Ostrom QT Gittleman H Truitt G Boscia A Kruchko C Barnholtz-Sloan JS. CBTRUS statistical report: primary brain and other central nervous system tumors diagnosed in the United States in 2011–2015. *Neuro Oncol.* (2018) 20:iv1–86. 10.1093/neuonc/noy131 30445539 PMC6129949

[70] Louis DN Perry A Reifenberger G von Deimling A Figarella-Branger D Cavenee WK The 2016 world health organization classification of tumors of the central nervous system: a summary. *Acta Neuropathol.* (2016) 131:803–20. 10.1007/s00401-016-1545-1 27157931

[71] Merchant TE Bendel AE Sabin ND Burger PC Shaw DW Chang E Conformal radiation therapy for pediatric ependymoma, chemotherapy for incompletely resected ependymoma, and observation for completely resected, supratentorial ependymoma. *J Clin Oncol.* (2019) 37:974–83. 10.1200/JCO.18.01765 30811284 PMC7186586

[72] Merchant TE Li C Xiong X Kun LE Boop F. Sanford. A prospective study of conformal radiation therapy for pediatric ependymoma. *Lancet Oncol.* (2009) 10:258–66. 10.1016/S1470-2045(08)70342-5.A 19274783 PMC3615425

[73] Tsang DS Burghen E Klimo P Boop FA Ellison DW Merchant TE. Outcomes after reirradiation for recurrent pediatric intracranial ependymoma. *Int J Radiat Oncol Biol Phys.* (2018) 100:507–15. 10.1016/j.ijrobp.2017.10.002 29229328

[74] Upadhyaya SA Robinson GW Onar-Thomas A Orr BA Billups CA Bowers DC Molecular grouping and outcomes of young children with newly diagnosed ependymoma treated on the multi-institutional SJYC07 trial. *Neuro Oncol.* (2019) 21:1319–30. 10.1093/neuonc/noz069 30976811 PMC6784269

[75] Zapotocky M Beera K Adamski J Laperierre N Guger S Janzen L Survival and functional outcomes of molecularly defined childhood posterior fossa ependymoma: cure at a cost. *Cancer.* (2019) 125:1867–76. 10.1002/cncr.31995 30768777 PMC6508980

[76] Pajtler KW Witt H Sill M Jones DTW Hovestadt V Kratochwil F Molecular classification of ependymal tumors across all CNS compartments, histopathological grades, and age groups. *Cancer Cell.* (2015) 27:728–43. 10.1016/j.ccell.2015.04.002 25965575 PMC4712639

[77] Yeung JT Hamilton RL Okada H Jakacki RI Pollack IF. Increased expression of tumor-associated antigens in pediatric and adult ependymomas: implication for vaccine therapy. *J Neurooncol.* (2013) 111:103–11. 10.1007/s11060-012-0998-x 23179498 PMC3546121

[78] Zhang JG Kruse CA Driggers L Hoa N Wisoff J Allen JC Tumor antigen precursor protein profiles of adult and pediatric brain tumors identify potential targets for immunotherapy. *J Neurooncol.* (2008) 88:65–76. 10.1007/s11060-008-9534-4 18259692 PMC4005736

[79] Witt H Mack SC Ryzhova M Bender S Sill M Isserlin R Delineation of two clinically and molecularly distinct subgroups of posterior fossa ependymoma. *Cancer Cell.* (2011) 20:143–57. 10.1016/j.ccr.2011.07.007 21840481 PMC4154494

[80] Griesinger AM Josephson RJ Donson AM Levy JMM Amani V Birks DK Interleukin-6/STAT3 pathway signaling drives an inflammatory phenotype in group a ependymoma. *Cancer Immunol Res.* (2015) 3:1165–74. 10.1158/2326-6066.CIR-15-0061 25968456 PMC4596749

[81] Witt DA Donson AM Amani V Moreira DC Sanford B Hoffman LM Specific expression of PD-L1 in RELA-fusion supratentorial ependymoma: implications for PD-1-targeted therapy. *Pediatr Blood Cancer.* (2018) 65:e26960. 10.1002/pbc.26960 29350470 PMC5867234

[82] Griesinger AM Birks DK Donson AM Amani V Hoffman LM Waziri A Characterization of distinct immunophenotypes across pediatric brain tumor types. *J Immunol.* (2013) 191:4880–8. 10.4049/jimmunol.1301966 24078694 PMC3827919

[83] Hoffman LM Donson AM Nakachi I Griesinger AM Birks DK Amani V Molecular sub-group-specific immunophenotypic changes are associated with outcome in recurrent posterior fossa ependymoma. *Acta Neuropathol.* (2014) 127:731–45. 10.1007/s00401-013-1212-8 24240813 PMC3988227

[84] Buscariollo DL Park HS Roberts KB Yu JB. Survival outcomes in atypical teratoid rhabdoid tumor for patients undergoing radiotherapy in a Surveillance, Epidemiology, and End Results analysis. *Cancer.* (2012) 118:4212–9. 10.1002/cncr.27373 22213196

[85] Lafay-Cousin L Hawkins C Carret AS Johnston D Zelcer S Wilson B Central nervous system atypical teratoid rhabdoid tumours: the canadian paediatric brain tumour consortium experience. *Eur J Cancer.* (2012) 48:353–9. 10.1016/j.ejca.2011.09.005 22023887

[86] Dufour C Beaugrand A Le Deley MC Bourdeaut F André N Leblond P Clinicopathologic prognostic factors in childhood atypical teratoid and rhabdoid tumor of the central nervous system: a multicenter study. *Cancer.* (2012) 118:3812–21. 10.1002/cncr.26684 22180295

[87] Zaky W Dhall G Ji L Haley K Allen J Atlas M Intensive induction chemotherapy followed by myeloablative chemotherapy with autologous hematopoietic progenitor cell rescue for young children newly-diagnosed with central nervous system atypical teratoid/rhabdoid tumors: the head start III experience. *Pediatr Blood Cancer.* (2014) 61:95–101. 10.1002/pbc.24648 23934933

[88] Chi SN Zimmerman MA Yao X Cohen KJ Burger P Biegel JA Intensive multimodality treatment for children with newly diagnosed CNS atypical teratoid rhabdoid tumor. *J Clin Oncol.* (2009) 27:385–9. 10.1200/JCO.2008.18.7724 19064966 PMC2645855

[89] Nicolaides T Tihan T Horn B Biegel J Prados M Banerjee A. High-dose chemotherapy and autologous stem cell rescue for atypical teratoid/rhabdoid tumor of the central nervous system. *J Neurooncol.* (2010) 98:117–23. 10.1007/s11060-009-0071-6 19936623 PMC2880232

[90] Reddy AT Strother DR Judkins AR Burger PC Pollack IF Krailo MD Efficacy of high-dose chemotherapy and three-dimensional conformal radiation for atypical teratoid/rhabdoid tumor: a report from the children’s oncology group trial ACNS0333. *J Clin Oncol.* (2020) 38:1175–85. 10.1200/JCO.19.01776 32105509 PMC7145589

[91] Biswas A Kashyap L Kakkar A Sarkar C Julka PK. Atypical teratoid/rhabdoid tumors: challenges and search for solutions. *Cancer Manag Res.* (2016) 8:115–25. 10.2147/CMAR.S83472 27695363 PMC5033212

[92] Torchia J Golbourn B Feng S Ho KC Sin-Chan P Vasiljevic A Integrated (epi)-genomic analyses identify subgroup-specific therapeutic targets in CNS rhabdoid tumors. *Cancer Cell.* (2016) 30:891–908. 10.1016/j.ccell.2016.11.003 27960086 PMC5500911

[93] Kram D Henderson J Baig M Chakraborty D Gardner M Biswas S Embryonal tumors of the central nervous system in children: the era of targeted therapeutics. *Bioengineering.* (2018) 5:78. 10.3390/bioengineering5040078 30249036 PMC6315657

[94] Torchia J Picard D Lafay-Cousin L Hawkins CE Kim SK Letourneau L Molecular subgroups of atypical teratoid rhabdoid tumours in children: an integrated genomic and clinicopathological analysis. *Lancet Oncol.* (2015) 16:569–82. 10.1016/S1470-2045(15)70114-2 25882982

[95] Coccé MC Lubieniecki F Kordes U Alderete D Gallego MS. A complex karyotype in an atypical teratoid/rhabdoid tumor: case report and review of the literature. *J Neurooncol.* (2011) 104:375–80. 10.1007/s11060-010-0478-0 21127945

[96] Johann PD Erkek S Zapatka M Kerl K Buchhalter I Hovestadt V Atypical teratoid/rhabdoid tumors are comprised of three epigenetic subgroups with distinct enhancer landscapes. *Cancer Cell.* (2016) 29:379–93. 10.1016/j.ccell.2016.02.001 26923874

[97] Batlle E Clevers H. Cancer stem cells revisited. *Nat Med.* (2017) 23:1124–34. 10.1038/nm.4409 28985214

[98] Golan H Shukrun R Caspi R Vax E Pode-Shakked N Goldberg S In vivo expansion of cancer stemness affords novel cancer stem cell targets: malignant rhabdoid tumor as an example. *Stem Cell Rep.* (2018) 11:795–810. 10.1016/j.stemcr.2018.07.010 30122444 PMC6135722

[99] Qi Y Zhao W Li M Shao M Wang J Sui H High C-X-C motif chemokine 5 expression is associated with malignant phenotypes of prostate cancer cells via autocrine and paracrine pathways. *Int J Oncol.* (2018) 53:358–70. 10.3892/ijo.2018.4388 29749439

[100] Gijsbers K Gouwy M Struyf S Wuyts A Proost P Opdenakker G GCP-2/CXCL6 synergizes with other endothelial cell-derived chemokines in neutrophil mobilization and is associated with angiogenesis in gastrointestinal tumors. *Exp Cell Res.* (2005) 303:331–42. 10.1016/j.yexcr.2004.09.027 15652347

[101] Linge HM Collin M Nordenfelt P Mörgelin M Malmsten M Egesten A. The human CXC chemokine granulocyte chemotactic protein 2 (GCP-2)/CXCL6 possesses membrane-disrupting properties and is antibacterial. *Antimicrob Agents Chemother.* (2008) 52:2599–607. 10.1128/AAC.00028-08 18443119 PMC2443903

[102] Schmidt T Samaras P Frejno M Gessulat S Barnert M Kienegger H ProteomicsDB. *Nucleic Acids Res.* (2018) 46:D1271–81. 10.1093/nar/gkx1029 29106664 PMC5753189

[103] Wilhelm M Schlegl J Hahne H Gholami AM Lieberenz M Savitski MM Mass-spectrometry-based draft of the human proteome. *Nature.* (2014) 509:582–7. 10.1038/nature13319 24870543

[104] Katoh M. Antibody-drug conjugate targeting protein tyrosine kinase 7, a receptor tyrosine kinase-like molecule involved in WNT and vascular endothelial growth factor signaling: effects on cancer stem cells, tumor microenvironment and whole-body homeostasis. *Ann Transl Med.* (2017) 5:462. 10.21037/atm.2017.09.11 29285495 PMC5733325

[105] Messerli SM Hoffman MM Gnimpieba EZ Bhardwaj RD. Therapeutic targeting of PTK7 is cytotoxic in atypical teratoid rhabdoid tumors. *Mol Cancer Res.* (2017) 15:973–83. 10.1158/1541-7786.MCR-16-0432 28442586 PMC5594561

[106] Damelin M Bankovich A Bernstein J Lucas J Chen L Williams S A PTK7-targeted antibody-drug conjugate reduces tumor-initiating cells and induces sustained tumor regressions. *Sci Transl Med.* (2017) 9:eaag2611. 10.1126/scitranslmed.aag2611 28077676

[107] Yang YP Nguyen PNN Ma HI Ho WJ Chen YW Chien Y Tumor mesenchymal stromal cells regulate cell migration of atypical teratoid rhabdoid tumor through exosome-mediated miR155/SMARCA4 pathway. *Cancers (Basel).* (2019) 11:720. 10.3390/cancers11050720 31137686 PMC6563126

[108] Lu JQ Wilson BA Yong VW Pugh J Mehta V. Immune cell infiltrates in atypical teratoid/rhabdoid tumors. *Can J Neurol Sci.* (2012) 39:605–12. 10.1017/S031716710001533X 22931701

[109] Melcher V Graf M Interlandi M Moreno N de Faria FW Kim SN Macrophage-tumor cell interaction promotes ATRT progression and chemoresistance. *Acta Neuropathol.* (2020) 139:913–36. 10.1007/s00401-019-02116-7 31848709

[110] Jones C Karajannis MA Jones DTW Kieran MW Monje M Baker SJ Pediatric high-grade glioma: biologically and clinically in need of new thinking. *Neuro Oncol.* (2016) 19:now101. 10.1093/neuonc/now101 27282398 PMC5464243

[111] Schwartzentruber J Korshunov A Liu X-Y Jones DTW Pfaff E Jacob K Driver mutations in histone H3.3 and chromatin remodelling genes in paediatric glioblastoma. *Nature.* (2012) 482:226–31. 10.1038/nature10833 22286061

[112] Wu G Broniscer A McEachron TA Lu C Paugh BS Becksfort J Somatic histone H3 alterations in pediatric diffuse intrinsic pontine gliomas and non-brainstem glioblastomas. *Nat Genet.* (2012) 44:251–3. 10.1038/ng.1102 22286216 PMC3288377

[113] Tate MC Lindquist RA Nguyen T Sanai N Barkovich AJ Huang EJ Postnatal growth of the human pons: a morphometric and immunohistochemical analysis. *J Comp Neurol.* (2015) 523:449–62. 10.1002/cne.23690 25307966 PMC4270924

[114] Braunstein S Raleigh D Bindra R Mueller S Haas-Kogan D. Pediatric high-grade glioma: current molecular landscape and therapeutic approaches. *J Neurooncol.* (2017) 134:541–9. 10.1007/s11060-017-2393-0 28357536

[115] Cohen KJ Pollack IF Zhou T Buxton A Holmes EJ Burger PC Temozolomide in the treatment of high-grade gliomas in children: a report from the Children’s Oncology Group. *Neuro Oncol.* (2011) 13:317–23. 10.1093/neuonc/noq191 21339192 PMC3064602

[116] Khuong-Quang D-A Buczkowicz P Rakopoulos P Liu X-Y Fontebasso AM Bouffet E K27M mutation in histone H3.3 defines clinically and biologically distinct subgroups of pediatric diffuse intrinsic pontine gliomas. *Acta Neuropathol.* (2012) 124:439–47. 10.1007/s00401-012-0998-0 22661320 PMC3422615

[117] Wolff JE Rytting ME Vats TS Zage PE Ater JL Woo S Treatment of recurrent diffuse intrinsic pontine glioma: the MD anderson cancer center experience. *J Neurooncol.* (2012) 106:391–7. 10.1007/s11060-011-0677-3 21858608 PMC3990187

[118] Susheela SP Revannasiddaiah S Muzumder S Mallarajapatna G Kallur K Basavalingaiah AS. Re-irradiation with hypo-fractionated stereotactic robotic radiotherapy for salvage in adult patients with brainstem glioma. *Ecancermedicalscience.* (2013) 7:366. 10.3332/ecancer.2013.366 24171050 PMC3805143

[119] Gwak H-S Park HJ. Developing chemotherapy for diffuse pontine intrinsic gliomas (DIPG). *Crit Rev Oncol Hematol.* (2017) 120:111–9. 10.1016/j.critrevonc.2017.10.013 29198324

[120] Truffaux N Philippe C Paulsson J Andreiuolo F Guerrini-Rousseau L Cornilleau G Preclinical evaluation of dasatinib alone and in combination with cabozantinib for the treatment of diffuse intrinsic pontine glioma. *Neuro Oncol.* (2015) 17:953–64. 10.1093/neuonc/nou330 25534822 PMC5654348

[121] Long W Yi Y Chen S Cao Q Zhao W Liu Q. Potential new therapies for pediatric diffuse intrinsic pontine glioma. *Front Pharmacol.* (2017) 8:495. 10.3389/fphar.2017.00495 28790919 PMC5525007

[122] Pollack IF Hamilton RL James CD Finkelstein SD Burnham J Yates AJ Rarity of PTEN deletions and EGFR amplification in malignant gliomas of childhood: results from the Children’s Cancer Group 945 cohort. *J Neurosurg Pediatr.* (2006) 105:418–24. 10.3171/ped.2006.105.5.418 17328268

[123] Nakamura M Shimada K Ishida E Higuchi T Nakase H Sakaki T Molecular pathogenesis of pediatric astrocytic tumors1. *Neuro Oncol.* (2007) 9:113–23. 10.1215/15228517-2006-036 17327574 PMC1871665

[124] Bredel M Pollack IF Hamilton RL James CD. Epidermal growth factor receptor expression and gene amplification in high-grade non-brainstem gliomas of childhood. *Clin Cancer Res.* (1999) 5:1786–92. 10430083

[125] Wolff JEA Driever PH Erdlenbruch B Kortmann RD Rutkowski S Pietsch T Intensive chemotherapy improves survival in pediatric high-grade glioma after gross total resection: results of the HIT-gBM-c protocol. *Cancer.* (2010) 116:705–12. 10.1002/cncr.24730 19957326

[126] Donaldson SS Laningham F Fisher PG. Advances toward an understanding of brainstem gliomas. *J Clin Oncol.* (2006) 24:1266–72. 10.1200/JCO.2005.04.6599 16525181

[127] Paugh BS Qu C Jones C Liu Z Adamowicz-Brice M Zhang J Integrated molecular genetic profiling of pediatric high-grade gliomas reveals key differences with the adult disease. *J Clin Oncol.* (2010) 28:3061–8. 10.1200/JCO.2009.26.7252 20479398 PMC2903336

[128] Paugh BS Broniscer A Qu C Miller CP Zhang J Tatevossian RG Genome-wide analyses identify recurrent amplifications of receptor tyrosine kinases and cell-cycle regulatory genes in diffuse intrinsic pontine glioma. *J Clin Oncol.* (2011) 29:3999–4006. 10.1200/JCO.2011.35.5677 21931021 PMC3209696

[129] Buczkowicz P Hoeman C Rakopoulos P Pajovic S Letourneau L Dzamba M Genomic analysis of diffuse intrinsic pontine gliomas identifies three molecular subgroups and recurrent activating ACVR1 mutations. *Nat Genet.* (2014) 46:451–6. 10.1038/ng.2936 24705254 PMC3997489

[130] Jin L Ge H Long Y Yang C Chang Y Mu L CD70, a novel target of CAR T-cell therapy for gliomas. *Neuro Oncol.* (2018) 20:55–65. 10.1093/neuonc/nox116 28651374 10.1093/neuonc/nox116 PMC5761579

[131] Shiina S Ohno M Ohka F Kuramitsu S Yamamichi A Kato A CAR T cells targeting podoplanin reduce orthotopic glioblastomas in mouse brains. *Cancer Immunol Res.* (2016) 4:259–68. 10.1158/2326-6066.CIR-15-0060 26822025

[132] Ahmed N Brawley V Hegde M Bielamowicz K Kalra M Landi D HER2-specific chimeric antigen receptor–modified virus-specific T cells for progressive glioblastoma: a phase 1 dose-escalation trial. *JAMA Oncol.*(2017) 3:1094–101. 10.1001/jamaoncol.2017.0184 28426845 PMC5747970

[133] Lee Y Martin-Orozco N Zheng P Li J Zhang P Tan H Inhibition of the B7-H3 immune checkpoint limits tumor growth by enhancing cytotoxic lymphocyte function. *Cell Res.* (2017) 27:1034–45. 10.1038/cr.2017.90 28685773 PMC5539354

[134] Prasad DVR Nguyen T Li Z Yang Y Duong J Wang Y Murine B7-H3 is a negative regulator of T cells. *J Immunol.* (2004) 173:2500–6. 10.4049/jimmunol.173.4.2500 15294965

[135] Du H Hirabayashi K Ahn S Kren NP Montgomery SA Wang X Antitumor responses in the absence of toxicity in solid tumors by targeting B7-H3 via chimeric antigen receptor T cells. *Cancer Cell.* (2019) 35:221–37.e8. 10.1016/j.ccell.2019.01.002 30753824 PMC6645919

[136] Chapoval AI Ni J Lau JS Wilcox RA Flies DB Liu D B7-H3: a costimulatory molecule for T cell activation and IFN-γ production. *Nat Immunol.* (2001) 2:269–74. 10.1038/85339 11224528

[137] Zhou Z Luther N Ibrahim GM Hawkins C Vibhakar R Handler MH B7-H3, a potential therapeutic target, is expressed in diffuse intrinsic pontine glioma. *J Neurooncol.* (2013) 111:257–64. 10.1007/s11060-012-1021-2 23232807 PMC4700828

[138] Mount CW Majzner RG Sundaresh S Arnold EP Kadapakkam M Haile S Potent antitumor efficacy of anti-GD2 CAR T cells in H3-K27M+ diffuse midline gliomas. *Nat Med.* (2018) 24:572–9. 10.1038/s41591-018-0006-x 29662203 PMC6214371

[139] Pule MA Savoldo B Myers GD Rossig C Russell HV Dotti G Virus-specific T cells engineered to coexpress tumor-specific receptors: persistence and antitumor activity in individuals with neuroblastoma. *Nat Med.* (2008) 14:1264–70. 10.1038/nm.1882 18978797 PMC2749734

[140] Louis CU Savoldo B Dotti G Pule M Yvon E Myers GD Antitumor activity and long-term fate of chimeric antigen receptor-positive T cells in patients with neuroblastoma. *Blood.* (2011) 118:6050–6. 10.1182/blood-2011-05-354449 21984804 PMC3234664

[141] Heczey A Louis CU Savoldo B Dakhova O Durett A Grilley B CAR T cells administered in combination with lymphodepletion and PD-1 inhibition to patients with neuroblastoma. *Mol Ther.* (2017) 25:2214–24. 10.1016/J.YMTHE.2017.05.012 28602436 PMC5589058

[142] Thomas S Straathof K Himoudi N Anderson J Pule M. An optimized GD2-targeting retroviral cassette for more potent and safer cellular therapy of neuroblastoma and other cancers. *PLoS One.* (2016) 11:e0152196. 10.1371/journal.pone.0152196 27030986 PMC4816271

[143] Pollack IF Jakacki RI Butterfield LH Hamilton RL Panigrahy A Potter DM Antigen-specific immune responses and clinical outcome after vaccination with glioma-associated antigen peptides and polyinosinic-polycytidylic acid stabilized by lysine and carboxymethylcellulose in children with newly diagnosed malignant brainstem and N. *J Clin Oncol.* (2014) 32:2050–8. 10.1200/JCO.2013.54.0526 24888813 PMC4067943

[144] Hegde M Mukherjee M Grada Z Pignata A Landi D Navai SA Tandem CAR T cells targeting HER2 and IL13Rα2 mitigate tumor antigen escape. *J Clin Invest.* (2016) 126:3036–52. 10.1172/JCI83416 27427982 PMC4966331

[145] Brown CE Alizadeh D Starr R Weng L Wagner JR Naranjo A Regression of glioblastoma after chimeric antigen receptor T-cell therapy. *N Engl J Med.* (2016) 375:2561–9. 10.1056/NEJMoa1610497 28029927 PMC5390684

[146] Funato K Major T Lewis PW Allis CD Tabar V. Use of human embryonic stem cells to model pediatric gliomas with H3.3K27M histone mutation. *Science.* (2014) 346:1529–33. 10.1126/science.1253799 25525250 PMC4995593

[147] Monje M Mitra SS Freret ME Raveh TB Kim J Masek M Hedgehog-responsive candidate cell of origin for diffuse intrinsic pontine glioma. *Proc Natl Acad Sci USA.* (2011) 108:4453–8. 10.1073/pnas.1101657108 21368213 PMC3060250

[148] Sturm D Witt H Hovestadt V Khuong-Quang D-A Jones DTW Konermann C Hotspot mutations in H3F3A and IDH1 define distinct epigenetic and biological subgroups of glioblastoma. *Cancer Cell.* (2012) 22:425–37. 10.1016/J.CCR.2012.08.024 23079654

[149] Lebel C Gee M Camicioli R Wieler M Martin W Beaulieu C. Diffusion tensor imaging of white matter tract evolution over the lifespan. *Neuroimage.* (2012) 60:340–52. 10.1016/J.NEUROIMAGE.2011.11.094 22178809

[150] Patterson J Wongsurawat T Rodriguez A. A glioblastoma genomics primer for clinicians. *Med Res Arch.* (2020) 8:2034. 10.18103/mra.v8i2.2034 32258388 PMC7111506

[151] Sottoriva A Spiteri I Piccirillo SGM Touloumis A Collins VP Marioni JC Intratumor heterogeneity in human glioblastoma reflects cancer evolutionary dynamics. *Proc Natl Acad Sci USA.* (2013) 110:4009–14. 10.1073/pnas.1219747110 23412337 PMC3593922

[152] Hambardzumyan D Bergers G. Glioblastoma: defining tumor niches. *Trends Cancer.* (2015) 1:252–65. 10.1016/j.trecan.2015.10.009 27088132 PMC4831073

[153] Daginakatte GC Gutmann DH. Neurofibromatosis-1 (Nf1) heterozygous brain microglia elaborate paracrine factors that promote Nf1-deficient astrocyte and glioma growth. *Hum Mol Genet.* (2007) 16:1098–112. 10.1093/hmg/ddm059 17400655

[154] Pong WW Higer SB Gianino SM Emnett RJ Gutmann DH. Reduced microglial CX3CR1 expression delays neurofibromatosis-1 glioma formation. *Ann Neurol.* (2013) 73:303–8. 10.1002/ana.23813 23424002 PMC3608848

[155] Komohara Y Ohnishi K Kuratsu J Takeya M. Possible involvement of the M2 anti-inflammatory macrophage phenotype in growth of human gliomas. *J Pathol.* (2008) 216:15–24. 10.1002/path.2370 18553315

[156] Kluiver TA Alieva M van Vuurden DG Wehrens EJ Rios AC. Invaders exposed: understanding and targeting tumor cell invasion in diffuse intrinsic pontine glioma. *Front Oncol.* (2020) 10:92. 10.3389/fonc.2020.00092 32117746 PMC7020612

[157] Lieberman NAP DeGolier K Kovar HM Davis A Hoglund V Stevens J Characterization of the immune microenvironment of diffuse intrinsic pontine glioma: implications for development of immunotherapy. *Neuro Oncol.* (2019) 21:83–94. 10.1093/neuonc/noy145 30169876 PMC6303470

[158] Lin GL Nagaraja S Filbin MG Suvà ML Vogel H Monje M. Non-inflammatory tumor microenvironment of diffuse intrinsic pontine glioma. *Acta Neuropathol Commun.* (2018) 6:51. 10.1186/s40478-018-0553-x 29954445 PMC6022714

[159] Sturm D Bender S Jones DTW Lichter P Grill J Becher O Paediatric and adult glioblastoma: multiform (epi)genomic culprits emerge. *Nat Rev Cancer.* (2014) 14:92–107. 10.1038/nrc3655 24457416 PMC4003223

[160] Gore L Zugmaier G Handgretinger R Locatelli F Trippett TM Rheingold SR Cytological and molecular remissions with blinatumomab treatment in second or later bone marrow relapse in pediatric acute lymphoblastic leukemia (ALL). *J Clin Oncol.* (2013) 31:10007–10007. 10.1200/jco.2013.31.15_suppl.10007

[161] Frey NV Porter DL. Cytokine release syndrome with novel therapeutics for acute lymphoblastic leukemia. *Hematology.* (2016) 2016:567–72. 10.1182/asheducation-2016.1.567 27913530 PMC6142489

[162] Thakar MS Kearl TJ Malarkannan S. Controlling cytokine release syndrome to harness the full potential of CAR-based cellular therapy. *Front Oncol.* (2020) 9:1529. 10.3389/fonc.2019.01529 32076597 PMC7006459

[163] Maude SL Barrett D Teachey DT Grupp SA. Managing cytokine release syndrome associated with novel T cell-engaging therapies. *Cancer J.* (2014) 20:119–22. 10.1097/PPO.0000000000000035 24667956 PMC4119809

[164] Grupp SA Kalos M Barrett D Aplenc R Porter DL Rheingold SR Chimeric antigen receptor–modified T cells for acute lymphoid leukemia. *N Engl J Med.* (2013) 368:1509–18. 10.1056/NEJMoa1215134 23527958 PMC4058440

[165] Teachey DT Rheingold SR Maude SL Zugmaier G Barrett DM Seif AE Cytokine release syndrome after blinatumomab treatment related to abnormal macrophage activation and ameliorated with cytokine-directed therapy. *Blood.* (2013) 121:5154–7. 10.1182/blood-2013-02-485623 23678006 PMC4123427

[166] Zhou X Dotti G Krance RA Martinez CA Naik S Kamble RT Inducible caspase-9 suicide gene controls adverse effects from alloreplete T cells after haploidentical stem cell transplantation. *Blood.* (2015) 125:4103–13. 10.1182/blood-2015-02-628354 25977584 PMC4481597

[167] Gargett T Brown MP. The inducible caspase-9 suicide gene system as a “safety switch” to limit on-target, off-tumor toxicities of chimeric antigen receptor T cells. *Front Pharmacol.* (2014) 5:235. 10.3389/fphar.2014.00235 25389405 PMC4211380

[168] Brudno JN Kochenderfer JN. Recent advances in CAR T-cell toxicity: Mechanisms, manifestations and management. *Blood Rev.* (2019) 34:45–55. 10.1016/j.blre.2018.11.002 30528964 PMC6628697

[169] Lee DW Santomasso BD Locke FL Ghobadi A Turtle CJ Brudno JN ASTCT consensus grading for cytokine release syndrome and neurologic toxicity associated with immune effector cells. *Biol Blood Marrow Transplant.* (2019) 25:625–38. 10.1016/j.bbmt.2018.12.758 30592986 PMC12180426

[170] Yakoub-Agha I Chabannon C Bader P Basak GW Bonig H Ciceri F Management of adults and children undergoing chimeric antigen receptor T-cell therapy: Best practice recommendations of the European Society for Blood and Marrow Transplantation (EBMT) and the Joint Accreditation Committee of ISCT and EBMT (JACIE). *Haematologica.* (2020) 105:297–316. 10.3324/haematol.2019.229781 31753925 PMC7012497

[171] Grigor EJM Fergusson D Kekre N Montroy J Atkins H Seftel MD Risks and benefits of chimeric antigen receptor T-cell (CAR-T) therapy in cancer: a systematic review and meta-analysis. *Transfus Med Rev.* (2019) 33:98–110. 10.1016/j.tmrv.2019.01.005 30948292

[172] Graber JJ Kesari S. Leptomeningeal metastases. *Curr Treat Options Oncol.* (2018) 19:3. 10.1007/s11864-018-0518-0 29362920

[173] O’Rourke DM Nasrallah MP Desai A Melenhorst JJ Mansfield K Morrissette JJD A single dose of peripherally infused EGFRvIII-directed CAR T cells mediates antigen loss and induces adaptive resistance in patients with recurrent glioblastoma. *Sci Transl Med.* (2017) 9:eaaa0984. 10.1126/scitranslmed.aaa0984 28724573 PMC5762203

[174] Topalian SL Hodi FS Brahmer JR Gettinger SN Smith DC McDermott DF Safety, activity, and immune correlates of anti–PD-1 antibody in cancer. *N Engl J Med.* (2012) 366:2443–54. 10.1056/NEJMoa1200690 22658127 PMC3544539

[175] Okada H Weller M Huang R Finocchiaro G Gilbert MR Wick W Immunotherapy response assessment in neuro-oncology: a report of the RANO working group. *Lancet Oncol.* (2015) 16:e534–42. 10.1016/S1470-2045(15)00088-1 26545842 PMC4638131

[176] Eisele SC Wen PY Lee EQ. Assessment of brain tumor response: RANO and its offspring. *Curr Treat Options Oncol.* (2016) 17:35. 10.1007/s11864-016-0413-5 27262709

[177] Topalian SL Sznol M McDermott DF Kluger HM Carvajal RD Sharfman WH Survival, durable tumor remission, and long-term safety in patients with advanced melanoma receiving nivolumab. *J Clin Oncol.* (2014) 32:1020–30. 10.1200/JCO.2013.53.0105 24590637 PMC4811023

[178] Okada H Kalinski P Ueda R Hoji A Kohanbash G Donegan TE Induction of CD8+ T-cell responses against novel glioma-associated antigen peptides and clinical activity by vaccinations with {alpha}-type 1 polarized dendritic cells and polyinosinic-polycytidylic acid stabilized by lysine and carboxymethylcellulose in. *J Clin Oncol.* (2011) 29:330–6. 10.1200/JCO.2010.30.7744 21149657 PMC3056467

[179] Wolchok JD Hoos A O’Day S Weber JS Hamid O Lebbe C Guidelines for the evaluation of immune therapy activity in solid tumors: immune-related response criteria. *Clin Cancer Res.* (2009) 15:7412–20. 10.1158/1078-0432.CCR-09-1624 19934295

[180] Kruit WHJ van Ojik HH Brichard VG Escudier B Dorval T Dréno B Phase 1/2 study of subcutaneous and intradermal immunization with a recombinant MAGE-3 protein in patients with detectable metastatic melanoma. *Int J Cancer.* (2005) 117:596–604. 10.1002/ijc.21264 15945101

[181] Van Baren N Bonnet M-C Dréno B Khammari A Dorval T Piperno-Neumann S Tumoral and immunologic response after vaccination of melanoma patients with an ALVAC virus encoding MAGE antigens recognized by T cells. *J Clin Oncol.* (2005) 23:9008–21. 10.1200/JCO.2005.08.375 16061912

[182] He Q Jiang X Zhou X Weng J. Targeting cancers through TCR-peptide/MHC interactions. *J Hematol Oncol.* (2019) 12:1–17. 10.1186/s13045-019-0812-8 31852498 PMC6921533

[183] Habib R Nagrial A Micklethwaite K Gowrishankar K. Chimeric antigen receptors for the tumour microenvironment. *Adv Exp Med Biol.* (2020) 1263:117–43. 10.1007/978-3-030-44518-8_8 32588326

[184] Schmitz F Wolf D Holderried TAW. The role of immune checkpoints after cellular therapy. *Int J Mol Sci.* (2020) 21:3650. 10.3390/ijms21103650 32455836 PMC7279282

[185] Habib S Tariq SM Tariq M. Chimeric antigen receptor-natural killer cells: the future of cancer immunotherapy. *Ochsner J.* (2019) 19:186–7. 10.31486/toj.19.0033 31528126 PMC6735593

[186] Ravanpay AC Gust J Johnson AJ Rolczynski LS Cecchini M Chang CA EGFR806-CAR T cells selectively target a tumor-restricted EGFR epitope in glioblastoma. *Oncotarget.* (2019) 10:7080–95. 10.18632/oncotarget.27389 31903167 PMC6925027

[187] Sibilia M Kroismayr R Lichtenberger BM Natarajan A Hecking M Holcmann M. The epidermal growth factor receptor: from development to tumorigenesis. *Differentiation.* (2007) 75:770–87. 10.1111/j.1432-0436.2007.00238.x 17999740

[188] Cheresh DA Pierschbacher MD Herzig MA Mujoo K. Disialogangliosides GD2 and GD3 are involved in the attachment of human melanoma and neuroblastoma cells to extracellular matrix proteins. *J Cell Biol.* (1986) 102:688–96. 10.1083/jcb.102.3.688 3005335 PMC2114134

[189] Brown CE Starr R Martinez C Aguilar B D’Apuzzo M Todorov I Recognition and killing of brain tumor stem-like initiating cells by CD8+ cytolytic T cells. *Cancer Res.* (2009) 69:8886–93. 10.1158/0008-5472.CAN-09-2687 19903840 PMC2789196

[190] Brown CE Starr R Aguilar B Shami AF Martinez C D’Apuzzo M Stem-like tumor-initiating cells isolated from IL13R 2 expressing gliomas are targeted and killed by IL13-zetakine-redirected T cells. *Clin Cancer Res.* (2012) 18:2199–209. 10.1158/1078-0432.CCR-11-1669 22407828 PMC3578382

[191] Yi Z Prinzing BL Cao F Gottschalk S Krenciute G. Optimizing EphA2-CAR T Cells for the Adoptive Immunotherapy of Glioma. *Mol Ther Methods Clin Dev.* (2018) 9:70–80. 10.1016/j.omtm.2018.01.009 29552579 PMC5852415

[192] Chow KKH Naik S Kakarla S Brawley VS Shaffer DR Yi Z T cells redirected to EphA2 for the immunotherapy of glioblastoma. *Mol Ther.* (2013) 21:629–37. 10.1038/mt.2012.210 23070117 PMC3589173

